# Preferred reporting items for studies mapping onto preference-based outcome measures: The MAPS statement

**DOI:** 10.1186/s12955-015-0305-6

**Published:** 2015-08-01

**Authors:** Stavros Petrou, Oliver Rivero-Arias, Helen Dakin, Louise Longworth, Mark Oppe, Robert Froud, Alastair Gray

**Affiliations:** Warwick Clinical Trials Unit, Warwick Medical School, University of Warwick, Coventry, CV4 7AL UK; National Perinatal Epidemiology Unit, Nuffield Department of Population Health, University of Oxford, Oxford, UK; Health Economics Research Centre, Nuffield Department of Population Health, University of Oxford, Oxford, UK; Health Economics Research Group, Brunel University London, Uxbridge, UK; EuroQol Research Foundation, Rotterdam, The Netherlands; Norges Helsehøyskole, Campus Kristiania, Oslo, Norway

## Abstract

‘Mapping’ onto generic preference-based outcome measures is increasingly being used as a means of generating health utilities for use within health economic evaluations. Despite publication of technical guides for the conduct of mapping research, guidance for the *reporting* of mapping studies is currently lacking. The MAPS (MApping onto Preference-based measures reporting Standards) statement is a new checklist, which aims to promote complete and transparent reporting of mapping studies. The primary audiences for the MAPS statement are researchers reporting mapping studies, the funders of the research, and peer reviewers and editors involved in assessing mapping studies for publication.

A *de novo* list of 29 candidate reporting items and accompanying explanations was created by a working group comprised of six health economists and one Delphi methodologist. Following a two-round, modified Delphi survey with representatives from academia, consultancy, health technology assessment agencies and the biomedical journal editorial community, a final set of 23 items deemed essential for transparent reporting, and accompanying explanations, was developed. The items are contained in a user friendly 23 item checklist. They are presented numerically and categorised within six sections, namely: (i) title and abstract; (ii) introduction; (iii) methods; (iv) results; (v) discussion; and (vi) other. The MAPS statement is best applied in conjunction with the accompanying MAPS explanation and elaboration document.

It is anticipated that the MAPS statement will improve the clarity, transparency and completeness of reporting of mapping studies. To facilitate dissemination and uptake, the MAPS statement is being co-published by eight health economics and quality of life journals, and broader endorsement is encouraged. The MAPS working group plans to assess the need for an update of the reporting checklist in five years’ time.

This statement was published jointly in *Applied Health Economics and Health Policy*, *Health and Quality of Life Outcomes*,* International Journal of Technology Assessment in Health Care*,* Journal of Medical Economics*, * Medical Decision Making*,* PharmacoEconomics*, and *Quality of Life Research*.

## Introduction

The process of ‘mapping’ onto generic preference-based outcome measures is increasingly being used as a means of generating health utilities for application within health economic evaluations [1]. Mapping involves the development and use of an algorithm (or algorithms) to predict the primary outputs of generic preference-based outcome measures, *i.e.* health utility values, using data on other indicators or measures of health. The source predictive measure may be a non-preference based indicator or measure of health outcome or, more exceptionally, a preference-based outcome measure that is not preferred by the local health technology assessment agency. The algorithm(s) can subsequently be applied to data from clinical trials, observational studies or economic models containing the source predictive measure(s) to predict health utility values in contexts where the target generic preference-based measure is absent. The predicted health utility values can then be analysed using standard methods for individual-level data (e.g. within a trial-based economic evaluation), or summarised for each health state within a decision-analytic model.

Over recent years there has been a rapid increase in the publication of studies that use mapping techniques to predict health utility values, and databases of published studies in this field are beginning to emerge [2]. Some authors [3] and agencies [4] concerned with technology appraisals have issued technical guides for the conduct of mapping research. However, guidance for the *reporting* of mapping studies is currently lacking. In keeping with health-related research more broadly [5], mapping studies should be reported fully and transparently to allow readers to assess the relative merits of the investigation [6]. Moreover, there may be significant opportunity costs associated with regulatory and reimbursement decisions for new technologies informed by misleading findings from mapping studies. This has led to the development of the MAPS (MApping onto Preference-based measures reporting Standards) reporting statement, which we summarise in this paper.

The aim of the MAPS reporting statement is to provide recommendations, in the form of a checklist of essential items, which authors should consider when reporting a mapping study. It is anticipated that the checklist will promote complete and transparent reporting by researchers. The focus, therefore, is on promoting the quality of reporting of mapping studies, rather than the quality of their conduct, although it is possible that the reporting statement will also indirectly enhance the methodological rigour of the research [7]. The MAPS reporting statement is primarily targeted at researchers developing mapping algorithms, the funders of the research, and peer reviewers and editors involved in the manuscript review process for mapping studies [5, 6]. In developing the reporting statement, the term ‘mapping’ is used to cover all approaches that predict the outputs of generic preference-based outcome measures using data on other indicators or measures of health, and encompasses related forms of nomenclature used by some researchers, such as ‘cross-walking’ or ‘transfer to utility’ [1, 8]. Similarly, the term ‘algorithm’ is used in its broadest sense to encompass statistical associations and more complex series of operations.

### The development of the MAPS statement

The development of the MAPS reporting statement was informed by recently published guidance for health research reporting guidelines [5] and broadly modelled other recent reporting guideline developments [9–14]. A working group comprised of six health economists (SP, ORA, HD, LL, MO, AG) and one Delphi methodologist (RF) was formed following a request from an academic journal to develop a reporting statement for mapping studies. One of the working group members (HD) had previously conducted a systematic review of studies mapping from clinical or health-related quality of life measures onto the EQ-5D [2]. Using the search terms from this systematic review, as well as other relevant articles and reports already in our possession, a broad search for reporting guidelines for mapping studies was conducted. This confirmed that no previous reporting guidance had been published. The working group members therefore developed a preliminary *de novo* list of 29 reporting items and accompanying explanations. Following further review by the working group members, this was subsequently distilled into a list of 25 reporting items and accompanying explanations.

Members of the working group identified 62 possible candidates for a Delphi panel from a pool of active researchers and stakeholders in this field. The candidates included individuals from academic and consultancy settings with considerable experience in mapping research, representatives from health technology assessment agencies that routinely appraise evidence informed by mapping studies, and biomedical journal editors. Health economists from the MAPS working group were included in the Delphi panel. A total of 48 of the 62 (77.4 %) individuals agreed to participate in a Delphi survey aimed at developing a minimum set of standard reporting requirements for mapping studies with an accompanying reporting checklist.

The Delphi panellists were sent a personalised link to a Web-based survey, which had been piloted by members of the working group. Non-responders were sent up to two reminders after 14 and 21 days. The panellists were anonymous to each other throughout the study and their identities were known only to one member of the working group. The panellists were invited to rate the importance of each of the 25 candidate reporting items identified by the working group on a 9-point rating scale (1, “not important”, to 9, “extremely important”); describe their confidence in their ratings (“not confident”, “somewhat confident” or “very confident”); comment on the candidate items and their explanations; suggest additional items for consideration by the panellists in subsequent rounds; and to provide any other general comments. The candidate reporting items were ordered within six sections: (i) title and abstract; (ii) introduction; (iii) methods; (iv) results; (v) discussion; and (vi) other. The panellists also provided information about their geographical area of work, gender, and primary and additional work environments. The data were imported into Stata (version 13; Stata-Corp, College Station, TX) for analysis.

A modified version of the Research ANd Development (RAND)/ University of California Los Angeles (UCLA) appropriateness method was used to analyse the round one responses [15]. This involved calculating the median score, the inter-percentile range (IPR) (30th and 70th), and the inter-percentile range adjusted for symmetry (IPRAS), for each item (_*i*_) being rated. The IPRAS includes a correction factor for asymmetric ratings, and panel disagreement was judged to be present in cases if IPR_*i*_ > IPRAS_*i*_ [15]. We modified the RAND/UCLA approach by asking panellists about ‘importance’ rather than ‘appropriateness’ *per se*. Assessment of importance followed the classic RAND/UCLA definitions, categorised simply as whether the median rating fell between 1 and 3 (unimportant), 4 and 6 (neither unimportant nor important), or 7 and 9 (important) [15].

The results of round one of the Delphi survey were reviewed at a face-to-face meeting of the working group. A total of 46 of the 48 (95.8 %) individuals who agreed to participate completed round one of the survey. Of the 25 items, 24 were rated as important, with one item (“Source of Funding”) rated as neither unimportant nor important. There was no evidence of disagreement on ratings of any items according to the RAND/UCLA method. These findings did not change when the responses of the MAPS working group were excluded. Based on the qualitative feedback received in round one, items describing “Modelling Approaches” and “Repeated Measurements” were merged, as were items describing “Model Diagnostics” and “Model Plausibility”. In addition, amendments to the wording of several recommendations and their explanations were made in the light of qualitative feedback from the panellists.

Panellists participating in round one were invited to participate in a second round of the Delphi survey. A summary of revisions made following round one was provided. This included a document in which revisions to each of the recommendations and explanations were displayed in the form of track changes. Panellists participating in round two were provided with group outputs (mean scores and their standard deviations, median scores and their IPRs, histograms and RAND/UCLA labels of importance and agreement level) summarising the round one results (and disaggregated outputs for the merged items). They were also able to view their own round one scores for each item (and disaggregated scores for the merged items). Panellists participating in round two were offered the opportunity to revise their rating of the importance of each of the items and informed that their rating from round one would otherwise hold. For the merged items, new ratings were solicited. Panellists participating in round two were also offered the opportunity to provide any further comments on each item or any further information that might be helpful to the group. Non-responders to the second round of the Delphi survey were sent up to two reminders after 14 and 21 days. The analytical methods for the round two data mirrored those for the first round.

The results of the second round of the Delphi survey were reviewed at a face-to-face meeting of the working group. A total of 39 of the 46 (84.8 %) panellists participating in round one completed round two of the survey. All 23 items included in the second round were rated as important with no evidence of disagreement on ratings of any items according to the RAND/UCLA method. Qualitative feedback from the panellists participating in round two led to minor modifications to wording of a small number of recommendations and their explanations. This was fed back to the round two respondents who were given a final opportunity to comment on the readability of the final set of recommendations and explanations. Based on these methods, a final consensus list of 23 reporting items was developed.

### The MAPS statement

The MAPS statement is a 23-item checklist of recommendations (Table 1) that we consider essential for complete and transparent reporting of studies that map onto generic preference-based outcome measures. The 23 reporting items are presented numerically and categorised within six sections, namely: (i) title and abstract (2 items); (ii) introduction (2 items); (iii) methods (9 items); (iv) results (6 items); (v) discussion (3 items); and (vi) other (1 item). The reporting of each item does not necessarily have to follow the order within the MAPS statement. Rather, what is important is that each recommendation is addressed either in the main body of the report or its appendices. Several biomedical journals have endorsed the MAPS statement. These include *Applied Health Economics and Health Policy*, *Health and Quality of Life Outcomes*, *International Journal of Technology Assessment in Health Care*, *Journal of Medical Economics*, *Medical Decision Making*, *PharmacoEconomics* and *Quality of Life Research*. We encourage other journals and research interest groups to endorse the MAPS statement and authors to adhere to its principles.

**Table 1:** Checklist of items to include when reporting a mapping study

Section/topic	Item number	Recommendation	Reported on page number/line number
Title and abstract			
Title	1	Identify the report as a study mapping between outcome measures. State the source measure(s) and generic, preference-based target measure(s) used in the study.	_____________
Abstract	2	Provide a structured abstract including, as applicable: objectives; methods, including data sources and their key characteristics, outcome measures used and estimation and validation strategies; results, including indicators of model performance; conclusions; and implications of key findings.	_____________
Introduction			
Study rationale	3	Describe the rationale for the mapping study in the context of the broader evidence base.	_____________
Study objective	4	Specify the research question with reference to the source and target measures used and the disease or population context of the study.	_____________
Methods			
Estimation sample	5	Describe how the estimation sample was identified, why it was selected, the methods of recruitment and data collection, and its location(s) or setting(s).	_____________
External validation sample	6	If an external validation sample was used, the rationale for selection, the methods of recruitment and data collection, and its location(s) or setting(s) should be described.	____________
Source and target measures	7	Describe the source and target measures and the methods by which they were applied in the mapping study.	_____________
Exploratory data analysis	8	Describe the methods used to assess the degree of conceptual overlap between the source and target measures.	_____________
Missing data	9	State how much data were missing and how missing data were handled in the sample(s) used for the analyses.	_____________
Modelling approaches	10	Describe and justify the statistical model(s) used to develop the mapping algorithm.	_____________
Estimation of predicted scores or utilities	11	Describe how predicted scores or utilities are estimated for each model specification.	_____________
Validation methods	12	Describe and justify the methods used to validate the mapping algorithm.	_____________
Measures of model performance	13	State and justify the measure(s) of model performance that determine the choice of the preferred model(s) and describe how these measures were estimated and applied.	_____________
Results			
Final sample size(s)	14	State the size of the estimation sample and any validation sample(s) used in the analyses (including both number of individuals and number of observations).	_____________
Descriptive information	15	Describe the characteristics of individuals in the sample(s) (or refer back to previous publications giving such information). Provide summary scores for source and target measures, and summarise results of analyses used to assess overlap between the source and target measures.	_____________
Model selection	16	State which model(s) is(are) preferred and justify why this(these) model(s) was(were) chosen.	_____________
Model coefficients	17	Provide all model coefficients and standard errors for the selected model(s). Provide clear guidance on how a user can calculate utility scores based on the outputs of the selected model(s).	_____________
Uncertainty	18	Report information that enables users to estimate standard errors around mean utility predictions and individual-level variability.	_____________
Model performance and face validity	19	Present results of model performance, such as measures of prediction accuracy and fit statistics for the selected model(s) in a table or in the text. Provide an assessment of face validity of the selected model(s).	_____________
Discussion			
Comparisons with previous studies	20	Report details of previously published studies developing mapping algorithms between the same source and target measures and describe differences between the algorithms, in terms of model performance, predictions and coefficients, if applicable.	_____________
Study limitations	21	Outline the potential limitations of the mapping algorithm.	_____________
Scope of applications	22	Outline the clinical and research settings in which the mapping algorithm could be used.	_____________
Other			
Additional information	23	Describe the source(s) of funding and non-monetary support for the study, and the role of the funder(s) in its design, conduct and report. Report any conflicts of interest surrounding the roles of authors and funders.	_____________

### The MAPS explanation and elaboration paper

In addition to the MAPS reporting statement, we have produced a supporting Explanation and Elaboration paper [16] modelled on those developed for other reporting guidelines [9–14]. The reporting items contained within the MAPS statement are best understood by referring to the information contained within this accompanying document. The Explanation and Elaboration paper provides exemplars of good reporting practice identified from the published literature for each reporting item. In addition, it provides a detailed explanation to accompany each recommendation, supported by a rationale and relevant evidence where available. The development of the Explanation and Elaboration paper was completed following several iterations produced by members of the working group, after which the examples and explanations were shared with the Delphi panellists for final revisions to improve readability and their approval. The Explanation and Elaboration paper also summarises the characteristics of the Delphi panellists and provides detailed statistics for item ratings at each Delphi round.

## Discussion

Over recent years, there has been a rapid increase in the publication of studies that use mapping techniques to predict health utility values. One recent review article identified ninety studies published up to the year 2013 reporting 121 mapping algorithms between clinical or health-related quality of life measures and the EQ-5D [2]. That review article excluded mapping algorithms targeted at other generic preference-based outcome measures that can generate health utilities, such as the SF-6D [17] and the Health Utilities Index (HUI) [18], which have been the target of numerous other mapping algorithms (e.g. [19–24, 1]). Moreover, the popularity of the mapping approach for estimating health utilities is unlikely to wane given the numerous contexts within health economic evaluation where primary data collection is challenging. However, mapping introduces additional uncertainty and collection of primary data with the preferred utility instrument is preferable.

The MAPS reporting statement was developed to provide recommendations, in the form of a checklist of essential items, which authors should consider when reporting mapping studies. Guidance for the reporting of mapping studies was not previously available in the literature. The overall aim of MAPS is to promote clarity, transparency and completeness of reporting of mapping studies. It is not intended to act as a methodological guide, nor as a tool for assessing the quality of study methodology. Rather, it aims to avoid misleading conclusions being drawn by readers, and ultimately policy makers, as a result of sub-optimal reporting. In keeping with other recent health research reporting guidelines, we have also produced an accompanying Explanation and Elaboration paper [16] to facilitate a deeper understanding of the 23 items contained within the MAPS reporting statement. That paper should hopefully act as a pedagogical framework for researchers reporting mapping studies.

The development of the MAPS reporting statement, and its Explanation and Elaboration document, was framed by recently published guidance for health research reporting guidelines [5]. The Delphi panel was composed of a multi-disciplinary, multi-national team of content experts and journal editors. The panel members included people experienced in conducting mapping studies; of the 84 researchers who were first authors on papers included in a recent review of EQ-5D mapping studies [2], 31 (36.9 %) were included as panellists. We have no evidence to believe that a larger panel would have altered the final set of recommendations. The Delphi methodologies that we applied included analytical approaches only recently adopted by developers of health reporting guidelines [15]. We are unable to assess whether a strict adherence to the MAPS checklist will increase the word counts of mapping reports. It is our view that the increasing use of online appendices by journals should permit comprehensive reporting even in the context of strict word limits for the main body of reports.

Evidence for other health research reporting guidelines suggests that reporting quality improved after the introduction of reporting checklists [25–27], although there is currently no empirical evidence that adoption of MAPS will improve the quality of reporting of mapping research. Future research planned by the MAPS working group will include a before and after evaluation of the benefits (and indeed possible adverse effects) of the introduction of the MAPS reporting statement. It will also be necessary to update the MAPS reporting statement in the future to address conceptual, methodological and practical advances in the field. Potential methodological advances that might be reflected in an update might include shifts towards more complex model specifications, better methods for dealing with uncertainty, and guidance on appropriate use of measures of prediction accuracy, such as mean absolute error (MAE) and mean square error (MSE). The MAPS working group plans to assess the need for an update of the reporting checklist in five years’ time.

In conclusion, this paper summarises a new reporting statement developed for studies that map onto generic preference-based outcome measures. We encourage health economic and quality of life journals to endorse MAPS, promote its use in peer review and update their editorial requirements and ‘Instructions to Authors’ accordingly.
